# Clinical significance, challenges and limitations in using artificial intelligence for electrocardiography-based diagnosis

**DOI:** 10.1186/s42444-022-00075-x

**Published:** 2022-10-01

**Authors:** Cheuk To Chung, Sharen Lee, Emma King, Tong Liu, Antonis A. Armoundas, George Bazoukis, Gary Tse

**Affiliations:** 1 Cardiac Electrophysiology Unit, Cardiovascular Analytics Group, Hong Kong, China; 2 Tianjin Key Laboratory of Ionic-Molecular Function of Cardiovascular Disease, Department of Cardiology, Tianjin Institute of Cardiology, Second Hospital of Tianjin Medical University, Tianjin, 300211 China; 3 Cardiovascular Research Center, Massachusetts General Hospital, Boston, MA USA; 4 Broad Institute, Massachusetts Institute of Technology, Cambridge, MA USA; 5 Department of Cardiology, Larnaca General Hospital, Inomenon Polition Amerikis, Larnaca, Cyprus; 6 Department of Basic and Clinical Sciences, University of Nicosia Medical School, 2414 Nicosia, Cyprus; 7 Kent and Medway Medical School, Canterbury, UK

**Keywords:** Electrocardiography, Artificial intelligence, Machine learning, Deep learning, Cardiovascular diseases

## Abstract

Cardiovascular diseases are one of the leading global causes of mortality. Currently, clinicians rely on their own analyses or automated analyses of the electrocardiogram (ECG) to obtain a diagnosis. However, both approaches can only include a finite number of predictors and are unable to execute complex analyses. Artificial intelligence (AI) has enabled the introduction of machine and deep learning algorithms to compensate for the existing limitations of current ECG analysis methods, with promising results. However, it should be prudent to recognize that these algorithms also associated with their own unique set of challenges and limitations, such as professional liability, systematic bias, surveillance, cybersecurity, as well as technical and logistical challenges. This review aims to increase familiarity with and awareness of AI algorithms used in ECG diagnosis, and to ultimately inform the interested stakeholders on their potential utility in addressing present clinical challenges.

## Introduction

An increasing number of research studies are currently investigating the potential use of artificial intelligence (AI) in different aspects of medicine [1–4]. In particular, there is increasing evidence supporting the role of AI in the field of electrocardiography (ECG). The ECG is a simple and inexpensive diagnostic tool that provides valuable guidance in assessing and monitoring cardiovascular diseases. Both performing and interpretating ECGs are skilled tasks requiring significant training and experience to achieve competency, and as such are susceptible to human error.

**Fig. 1:**
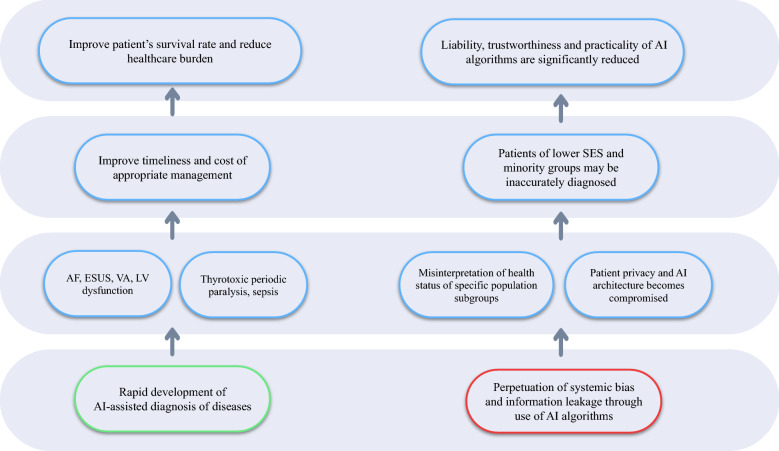
Flow diagram summarising the clinical significance, challenges, and limitations of using artificial intelligence (AI) for electrocardiography (ECG)-based diagnosis

Application of supervised and unsupervised machine learning (ML) algorithms in ECG analysis and interpretation has shown considerable promise [5]. Supervised ML such as Artificial Neural Networks (ANNs) and Support Vector Machine (SVM) can be trained to learn a classification function given a set of labelled data. Contrarily, unsupervised ML can detect potential correlations in data without labels [6]. In addition to ML, the recent advent of deep learning-based (DL) analysis of ECGs can assist clinicians in different clinical scenarios, such as cardiovascular diseases (arrhythmias, cardiomyopathies, valve diseases, etc.) and non-cardiovascular diseases, for diagnosis, prognosis, and risk stratification [7–12]. Instead of being fed with handcrafted vectors, on which ML algorithms rely, the DL approach uses the end-to-end learning strategy, which programmes the system to learn the necessary features from the raw data [13]. The benefit of deep neural networks is its ability to identify novel intervariable relationships independent of human-selected feature extraction, which can offer an enormous previously unrecognized insights in healthcare diagnosis and treatement [7]. This review aims to discuss the clinical significance and implications of the use of AI algorithms on ECG (AI-ECG) analysis and interpretation as well as the associated clinical challenges and limitations (Fig. 1).

## Clinical significance

### Cardiovascular disease

With regard to cardiovascular conditions, the use of AI-ECG algorithms for rhythm identification and ECG interpretation can be more accurate in interpretation than existing ECG software [14]. AI-based ECG analysis showed high accuracy in diagnosing hypertrophic cardiomyopathy [15], with an area under the receiver operating characteristic curve (AUC) of 0.98, sensitivity of 92% and specificity of 95%. Moreover, AI-based ECG analysis could also be used for screening first-degree relatives of patients with dilated cardiomyopathy [16]. For the rare catecholaminergic polymorphic ventricular tachycardia with apparently normal ECGs at rest [17, 18], subtle repolarization abnormalities may be detected by machine learning methods [19].

Energy waveform ECG analysis using ML techniques has also been successfully employed in identifying patients with left ventricular (LV) dysfunction, reducing the need for echocardiography exams in asymptomatic patients [20]. Similarly, AI-based analysis of ECGs was able to identify patients with dyspnea caused by LV systolic dysfunction, which outperformed the use of NT-proBNP as a marker of LV failure [21].

Another area for AI implementation is the identification of patients who may benefit from anticoagulation following an embolic stroke of unknown source (ESUS). A probability greater than 0.2, of underlying atrial fibrillation (AF) determined by AI-ECG analysis, in patients with ESUS was associated with increased AF detection during ambulatory cardiac rhythm monitoring [22]. Recent work has demonstrated that ML algorithms can detect AF by restitution analysis of normal sinus rhythm ECGs with k-nearest neighbour (k-NN) algorithm [23]. With respect to AF detection, traditionally handcrafted algorithms cannot provide a rich data representation as they are unable to accurately detect P-waves due to their low signal-to-noise ratio and overlapping with the T-waves, which reduces their performance [24]; by contrast, DL algorithms are able to “view” the complexity of the signal holistically by understanding the relationships that exist between parameters [24]. The presence of artifacts in ECG images may pose a challenge in the use of convolutional neural network (CNN) in image training. However, the tecent, introduction of recurrent neural networks (RNN) can further minimize potential bias and discriminate AF from normal sinus rhythm [25]. Consequently, if AI enhanced ECG analysis is implemented and adopted, it may save unnecessary inspection time and costs on follow up diagnostic tests or monitoring devices. While there is still limited supporting evidence for systematic screening for AF, as well as associated cost implications [26], targeted screening, systemic opportunistic screening or smartphone algorithms, may be a more cost-effective option when using AI-enhanced ECG systems. With an increasing consumer adoption of wearable healthcare technologies [27, 28], the incorporation of AI-enhanced algorithms for AF screening [29–32] would be expected to AF-related morbidities in the long-term [33, 34].

Regarding ventricular arrhythmias, AI analysis of ECGs can be used to identify the site of origin, aiding catheter ablation procedures and quality of care in the ICU [35, 36]. In the setting of valvular diseases, AI-based ECG analysis has been proposed as an accurate screening tool for the identification of patients with moderate to severe aortic stenosis in the community [37]. Furthermore, promising results have been proposed for the identification of mitral regurgitation using AI-based ECG [38]. ML algorithms have been implemented in the hospitalized patients to improve the quality of care. Specifically, most alarms in bedside monitors have been found to be false. ML algorithms have been implemented in this setting by reducing false alarms improving the quality of care in hospitalized patients [39, 40].

### Non-cardiovascular disease

Beyond the role of AI-based analysis of ECG data in the management of cardiovascular disease, AI can be used for the analysis of ECG data to aid the management of various non-cardiovascular diseases. AI has been used to identify the ECG-age of patients. Interestingly, it was found that patients with ECG-age more than eight years greater than their chronological age have higher mortality rates [9].

Sepsis can be identified by the application of DL algorithms on ECG data [41], while hyperglycemia has been identified by an ML algorithm using ECG data with an AUC of 0.945, sensitivity of 88%, and specificity of 85% [42]. ML-based ECG analysis has also been applied to the non-invasive evaluation of physiological parameters, including cardiovascular and cerebrovascular hemodynamics [43]. An AI-ECG system in combination with routine blood chemistries can aid the early diagnosis of thyrotoxic periodic paralysis, facilitating the timely initiation of appropriate management [9, 44]. Moreover, recent studies have also demonstrated the use of DL models and ECG to assess electrolyte imbalances [45]. This methodology is economical, and data can be easily obtained with wearables. Electrolyte imbalance is a marker for numerous endocrine diseases such as diabetes insipidus and hyperparathyroidism [46]. AI-based ECG analysis was also found to achieve a high negative predictive value for COVID-19 and, therefore, can be used as a rapid screening tool in identifying healthy individuals [47]. The performance of the aforementioned AI-ECG algorithms is summarized in Table 1. The variation in sensitivity and specificity may be attributed to variability in the cohort size of each study. As AI technology becomes more refined and accessible, it can offer increased diagnostic efficiency for a range of conditions, thus resulting in more prompt and efficient care.

**Table 1:** Summary of the different AI-ECG algorithms of included studies

Study	Year	Machine learning technique	AUC	Specificity (%)	Sensitivity (%)
Adedinsewo et al. [21]	2020	Convolutional neural network	0.890	87	74
Attia et al. [47]	2021	Convolutional and residual neural network	0.767	10.2	98
Cohen-Shelly et al. [37]	2021	Convolutional neural network	0.850	74	78
Cordeiro et al. [42]	2021	Deep neural network	0.945	85	87.6
Kwon et al. [41]	2021	Residual neural network	0.901	–	–
Kwon et al. [45]	2020	Convolutional neural network	0.873	–	–
Lin et al. [44]	2021	Convolutional neural network	0.986	69.2	88.9
Potter et al. [20]	2021	Random forest classifier	0.830	72	85
Rabinstein et al. [22]	2021	–	–	75	63
Shrivastava et al. [16]	2021	Convolutional neural network	0.955	44.8	98.8
Siontis et al. [15]	2021	Convolutional neural network	0.980	95	92

## Challenges and limitations

### Professional liability

Despite its promising potential, several issues must be addressed regarding the clinical implementation of AI. More importantly, a framework for successful implementation is mandatory [1, 48, 49]. With greater AI involvement in clinical decision-making, our previous perception of the dynamics of practitioners’ fiduciary relationship with their patients will be challenged. Another issue that has raised ethical concerns for AI implementation in clinical practice is physicians’ professional liability in the case of an incorrect decision [48, 50]. Inexperienced clinicians may blindly trust the diagnosis of AI algorithms. Consequently, a complication or medical malpractice may be further perplexed, since both healthcare professionals and AI developers are involved.

From a legal perspective, frameworks that apply to existing medical products may not apply to the liability of AI technology. Due to current medical malpractice laws, some healthcare professionals may be incentivized to use AI as a confirmatory tool rather than a sole diagnostic method to prevent liability issues [51]. When dealing with misuse or mismanagement, the concept of accountability is essential for the use of AI in clinical decision-making. Therefore, the indications and possible adverse effects in using an AI algorithm should be stated clearly by the manufacturing company, in a similar way to the other conventional medical products.

### Systemic bias

AI algorithms may be subjected to “learn” from biased data. This can be attributed to three main causes: model bias due to the overrepresentation of a majority class; model variance due to inadequate data for minority groups; and outcome noise caused by the undesired effect of unobserved variables [52]. As ECG features vary between races, the generalizability of DL algorithms could be affected by patient selection bias [53].

Also, due to the reliance on electronic health records, it is inevitable that some data will be missing or inaccessible from each data set. For example, there may be fewer data available for specific population subgroups, such as citizens of lower socioeconomic status, as they may have performed fewer diagnostic tests due to limited access to health services [54]. In such case, a system may misinterpret the lack of healthcare usage or a small sample size as a lower disease burden. Similarly, health records collected from wearables may skew the sample population towards socio-economically advantaged individuals [51].

The influence of bias on AI-ECG algorithms is manifested among others in two studies, aiming to detect aortic stenosis [55] and AF [56], in which the accuracy of disease detection is lower amongst populations with a lower disease prevalence. In the case of AF, the problem is exacerbated by the paroxysmal and often undetectable nature of AF, leading to inaccurate data and incorrect diagnosis of patients at baseline or follow-up. When data has been limited to either positive or negative labels, this results in a modest effect on the overestimation of test performance.

In research studies that employ small volumes of data, traditional statistical methods may be superior to ML, which performs better for large datasets [35]. Even when the representation of minority groups exists in a data set, insufficient sample size will be of little use to the development of AI algorithms, which require an exhaustive collection of health records. This leads to the phenomenon known as underestimation that leads algorithms to approximate mean trends in order to avoid overfitting [57]. AI-ECG algorithms may also be prone to overfitting, resulting in poor performance in test sets with limited generalizability [58].

Algorithms developed in university hospitals often sample data from a Western, youthful and high-income population subgroup [54]. Hence, this may lead to a skewed interpretation of available data rather than a holistic view of the whole population. Resultantly, data deficiency may exacerbate socioeconomic disparities. Interestingly, a study by Noseworthy et al*.* demonstrated that the performance of a DL AI algorithm in using ECGs to detect left ventricular ejection fraction ≤ 35% was not influenced by the variation of race, even though a homogenous population was used to develop the algorithm, which reflects the unpredictable relationship between race and algorithm systems [53].

However, such an issue may be alleviated if algorithms from single institutions could be amended and integrated with external data sets that represent more racially and socioeconomically diverse population subgroups. It is imperative that diversity exists not just in the sample population but also in developers, healthcare professionals and medical experts in order to ensure that different social groups are sufficiently represented and identify potential discriminatory aspects of the data analysis process.

### Surveillance and cybersecurity

Surveillance of the safety and accuracy of a specific algorithm should be ensured by the responsible authorities. Compared to other medical products, surveillance is more important in the case of AI algorithms due to system upgrades that may influence algorithm performance. It must be noted that some AI systems are designed to be dynamic and can re-calibrate through self-learning. Proactive protection of patient rights and protection of personal data should also be upheld. Among other issues, the increasing anthropomorphization of AI may also pose potential security risks and ethical concerns [59].

Finally, cybersecurity is another topic of interest, as AI algorithms can be a potential target for hacking [60, 61]. Almost all processing stages of the AI architecture, including the initial data inputs, human–machine teaming to the data-conditioning process, are susceptible to cyberattacks [59]. This is a unique type of cyber vulnerability compared to traditional information and technology systems, in which the hardware or software is generally the target of cyberattacks [59].

In that vein, there are four common types of attacks against AI systems [59]. Firstly, attackers can tamper with categorization models and adjust the outcome of the AI algorithm; secondly, data inputted into the AI can be altered by mixing erroneous data with actual data sets, which significantly impacts the AI training process; thirdly, through reverse engineering, attackers can perform multiple adversarial attacks and create knockoff systems; fourthly, backdoor attacks can be stealthily conducted by overriding existing classifications and infiltrating data sets through data “poisoning”. Subsequently, hacking can influence the performance of AI systems and can adversely affect patient outcomes. This makes the prevention of hacking a prerequisite for the implementation of these systems in clinical practice.

As it is inevitable that collaboration and exchange of data between different institutions is necessary to build large data sets required for AI model development [62], another concern pertinent to patient privacy is the use of anonymized data. As the size of the ECG data set increases, existing anonymization techniques may not be effective [63, 64]. Aside from medical information, the identification of demographic variables, socioeconomic class and religious status may contribute to discrimination of specific population subgroups [65]. Moreover, an ECG data ownership conflict may arise when the data are obtained from smartphone-based applications. Hence, it is crucial to establish regulations for all stakeholders and federated learning architectures to safeguard data sharing [62].

In recent years, the US Food and Drug Administration has advanced considerably in developing a rigorous regulatory framework for the medical use of AI [66]. For example, ML apps such as the Apple watch that pose a moderate to high risk to individual user safety have to comply to the regulatory requirements of the FDA [67].

Additionally, there have been considerable efforts towards developing cyberattack detection systems to protect the e-Health infrastucture. Son et al*.* developed an intelligent ECG monitoring system to detect arrythmia occurrence while incorporating anonymous identity schemes and signal scrambling to protect user privacy [68]. To preserve anonymity, a pseudonym was given to each user and a pseudo-random generator-based stream cipher was implemented to safeguard the ECG signal. The study also ensured to utilize a conventional public key cryptosystem to secure the transfer of sensing data from the sensor to the monitoring station.

### Technical challenges for incorporating AI-ECG into clinical practice

There are many technical challenges when attempting to incorporate and AI algorithms into clinical practice. The first is standardization of data. Variations in existing ECG input data types, storage formats and interpretation statements unavoidably limit the broad interoperability of ECG data [69]. These formats differ depending on whether the data pertain to resting, ambulatory, bedside or ECG from mobile devices that could cause miscommunication and discrepancies in the understanding of the data. Including standardized reporting statements increases the utility and consistency of ECG interpretation, AI algorithms need a large amount of high-quality data to provide accurate results. This is a major limitation for developing algorithms in the management of rare diseases with limited sample size. In addition, data that are either incomplete, heterogeneous, noisy or ambiguous poses an additional challenge as it could affect the quality of data [51]. Resultantly, this may lead to missing values, redundancy, or data sparsity [51]. However, the collaboration between centers and the development of multicenter registries can help to overcome this issue. Furthermore, the adoption of a unified ECG data format such as the Fast Healthcare Interoperability Resources could improve the aggregation of data, maximize the extraction of useful data and support all diagnostic modalities [70].

ECG data collection should be followed by their proper annotation. In a real-world setting, the population cannot always be well-phenotyped and the quality of ECG data may vary, which may lead to discrepancies between the published performance results of an algorithm and its performance in clinical practice. Consequently, ML algorithms should be used only for the specific population they have been trained for.

Another concern regarding AI-based analysis of ECG data is the interpretation and incorporation of the algorithm results into clinical practice. Beyond the testing and validation of a proposed algorithm, additional studies are needed to investigate its performance compared to existing diagnostic tools [71]. Therefore, clinicians must be trained on how to integrate information derived from AI algorithms into their practice, an aspect that is often overlooked in research papers. Overall, designing accessible AI curriculum and training programs may empower practitioners to effectively integrate AI in their clinical practice.

### Logistical challenges

Currently, technical variations in coding definitions, electronic health record systems, administrative procedures, laboratory equipment and clinical practice may limit the clinical utility of an algorithm [71]. A large portion of clinical studies have used ECG printouts, possibly because of a lack of access to digital files. If access is a problem, then re-digitalization using recognition software is needed before further application of machine learning techniques [69].

## Conclusion

In conclusion, AI-based analysis of ECG signals is expected to revolutionize healthcare diagnostic and prognostic services, in both cardiology and non-cardiology-related diseases. To ensure AI can safely elevate the quality of healthcare services, a framework that regulates the implementation of AI is mandatory. Scientists must be cognizant of the existing limitations of AI-based analysis of ECGs, such as potential bias and actively design interventions to safeguard undesirable outcomes. With such measures, AI can be implemented on a global scale for clinical practice. Ultimately, AI has the potential to offer data-driven clinical decision support systems.

## Data Availability

Not applicable.
